# Environmental Concentrations of Sulfonamides Can Alter Bacterial Structure and Induce Diatom Deformities in Freshwater Biofilm Communities

**DOI:** 10.3389/fmicb.2021.643719

**Published:** 2021-05-07

**Authors:** Laura Kergoat, Pascale Besse-Hoggan, Martin Leremboure, Jérémie Beguet, Marion Devers, Fabrice Martin-Laurent, Matthieu Masson, Soizic Morin, Amélie Roinat, Stéphane Pesce, Chloé Bonnineau

**Affiliations:** ^1^INRAE, UR RiverLy, Villeurbanne, France; ^2^Université Clermont Auvergne, CNRS, Sigma Clermont, Institut de Chimie de Clermont-Ferrand, Clermont–Ferrand, France; ^3^AgroSup Dijon, INRAE, Univ. Bourgogne Franche-Comté, Agroécologie, Dijon, France; ^4^INRAE, UR EABX, Cestas, France

**Keywords:** periphyton, antibiotic, teratogenicity, microbial ecotoxicology, sulfamethazine, sulfamethoxazole

## Abstract

Since the early 1920s, the intensive use of antibiotics has led to the contamination of the aquatic environment through diffuse sources and wastewater effluents. The antibiotics commonly found in surface waters include sulfamethoxazole (SMX) and sulfamethazine (SMZ), which belong to the class of sulfonamides, the oldest antibiotic class still in use. These antibiotics have been detected in all European surface waters with median concentrations of around 50 ng L^–1^ and peak concentrations of up to 4–6 μg L^–1^. Sulfonamides are known to inhibit bacterial growth by altering microbial production of folic acid, but sub-lethal doses may trigger antimicrobial resistance, with unknown consequences for exposed microbial communities. We investigated the effects of two environmentally relevant concentrations (500 and 5,000 ng L^–1^) of SMZ and SMX on microbial activity and structure of periphytic biofilms in stream mesocosms for 28 days. Measurement of sulfonamides in the mesocosms revealed contamination levels of about half the nominal concentrations. Exposure to sulfonamides led to slight, transitory effects on heterotrophic functions, but persistent effects were observed on the bacterial structure. After 4 weeks of exposure, sulfonamides also altered the autotrophs in periphyton and particularly the diversity, viability and cell integrity of the diatom community. The higher concentration of SMX tested decreased both diversity (Shannon index) and evenness of the diatom community. Exposure to SMZ reduced diatom species richness and diversity. The mortality of diatoms in biofilms exposed to sulfonamides was twice that in non-exposed biofilms. SMZ also induced an increase in diatom teratologies from 1.1% in non-exposed biofilms up to 3% in biofilms exposed to SMZ. To our knowledge, this is the first report on the teratological effects of sulfonamides on diatoms within periphyton. The increase of both diatom growth rate and mortality suggests a high renewal of diatoms under sulfonamide exposure. In conclusion, our study shows that sulfonamides can alter microbial community structures and diversity at concentrations currently present in the environment, with unknown consequences for the ecosystem. The experimental set-up presented here emphasizes the interest of using natural communities to increase the ecological realism of ecotoxicological studies and to detect potential toxic effects on non-target species.

## Introduction

The number of scientific articles on the occurrence and impact of pharmaceutical residues in the environment has greatly increased since the early 2000s (Daughton, 2016), illustrating a growing concern with the wide dissemination of these biologically active substances in the environment and their potential impact on ecosystems. Pharmaceutical residues can reach freshwater ecosystems directly from aquaculture, run-off from agricultural soils amended with organic matter or from the effluents of wastewater treatment plants, pharmaceutical manufacturing plants and animal husbandry (Halling-Sørensen et al., 1998; Paíga et al., 2016; Mandaric et al., 2017). Numerous monitoring studies have shown the wide distribution of pharmaceutical products and their transformation products in European freshwaters at concentrations ranging from ng L^–1^ up to μg L^–1^, with a high proportion of antibiotics (for more details, see the reviews of Carvalho and Santos, 2016; Szymañska et al., 2019).

The work reported here focuses on sulfonamides, one of the oldest antibiotic classes still in use for both animal and human health care. Sulfonamides are bacteriostatic compounds that inhibit the growth of microbial organisms (e.g., protozoa, fungi, certain bacteria; Straub, 2016) that rely on folate synthesis for the synthesis of purine and pyrimidine nucleotides (McDermott et al., 2003). The occurrence of sulfonamides in European surface waters is well documented (e.g., Kovalakova et al., 2020): in a review on environmental risk assessment of sulfamethoxazole, Straub (2016) found that in most studies, sulfamethoxazole concentrations in surface water were <286 ng L^–1^ with a median of 52 ng L^–1^ (*n* = 5,536 surface water samples). A few measured environmental concentrations reached higher levels, up to 4 μg L^–1^, such concentrations being mainly found in streams where wastewater treatment plant effluents were released (Straub, 2016). Sulfamethazine concentrations in European freshwaters are in the range of those observed for sulfamethoxazole (see Figure 1 in Charuaud et al., 2019). However, this antibiotic has also been found at concentrations of up to 6 μg L^–1^ in the Llobregat river (Spain) by Díaz-Cruz et al. (2008) and up to 12 μg L^–1^ in a Croatian river receiving industrial waste water (Bielen et al., 2017).

A major concern regarding the general contamination of freshwaters (and other environmental compartments) by sulfonamides (and other antibiotics) is the increasing occurrence of microbial antibiotic resistance in the environment (Martin-Laurent et al., 2019). At sub-lethal doses, antibiotics exert a selection pressure that will promote the emergence and development of antibiotic resistance (for a complete review on antibiotic resistance mechanisms, see McDermott et al., 2003). Resistance to sulfonamides is generally conferred by the acquisition of an enzyme insensitive to sulfonamides that restores the folic acid synthesis pathway (McDermott et al., 2003). This enzyme is encoded by *sul* genes (i.e., *sul1, sul2*, and *sul3*), generally found on a plasmid, which facilitates their dissemination by horizontal gene transfer across microbial communities (Sköld, 2000; Perreten and Boerlin, 2003). Antibiotic resistance genes are frequently detected in natural microbial communities (Martínez, 2008) in both periphytic biofilms (e.g., Di Cesare et al., 2017; Guo et al., 2018) and sediments (e.g., Marti et al., 2013; Czekalski et al., 2014; Calero-Cáceres et al., 2017) with higher levels being detected downstream from the discharge of effluents of wastewater treatment plants (Proia et al., 2016). The dissemination and occurrence of antibiotic resistance genes in aquatic ecosystems are likely to be a threat for environmental health and have led several authors to qualify antibiotic resistance genes as emerging contaminants (Sanderson et al., 2016; O’Flaherty and Cummins, 2017).

Besides their implication in the dissemination of antibiotic resistance genes, antibiotics could have a direct toxic impact on aquatic organisms. Ecotoxicity of sulfamethoxazole in a wide range of aquatic model species is well known, with 236 entries for NOEC (no observed effect concentration) in the ECOTOX database of US EPA (U.S. Environmental Protection Agency, 2019). Focusing on endpoints directly related to population dynamics (i.e., growth, reproduction, survival, etc.), sulfamethoxazole NOECs range from 5.9 μg L^–1^ for the cyanobacterium *Synechococcus leopoliensis* to 253 mg L^–1^ for the protist *Tetrahymena pyriformis* (reviewed by Straub, 2016). Information on sulfamethazine ecotoxicity is scarce, with only three entries available for NOEC in the ECOTOX database of US EPA. Yang et al. (2008) reported a NOEC of 1 mg L^–1^ for the growth of the microalga *Pseudokirchneriella subcapitata*. De Liguoro et al. (2009) reported a NOEC of 50 mg L^–1^ for the survival of the crustacean *Daphnia magna*. The distribution of species sensitivity toward sulfonamides underlines a high sensitivity of cyanobacteria and microbial communities to this antibiotic (Straub, 2016; Charuaud et al., 2019).

In aquatic environments, antibiotics are generally present at lower concentrations than those producing bactericidal or bacteriostatic effects. Even so, this does not rule out an ecotoxicological risk for chronically exposed communities. For example, sub-lethal concentrations of antibiotics may act as signaling molecules (e.g., “danger signal,” Moon et al., 2017) and trigger specific responses (e.g., biofilm formation, Linares et al., 2006) different from the effects of inhibitory concentrations (Fajardo and Martínez, 2008). Environmental concentrations of antibiotics might therefore affect natural microbial communities, with possible consequences for microbial diversity and functions and thereby for ecosystem processes supported by those communities.

Among aquatic microbial communities, periphytic biofilms are complex communities composed of bacteria, microalgae, fungi and protozoa, among other organisms. In lotic ecosystems, they play an essential role in important ecological processes including primary production and nutrient recycling (Battin et al., 2016). Previous studies have shown that environmental concentrations of antimicrobial agents and/or antibiotics can modify aquatic biofilm structure (e.g., triclosan in Lawrence et al., 2015) and function (e.g., tetracycline on bacterial productivity, Quinlan et al., 2011; triclosan on phosphate uptake capacity, Proia et al., 2013b). Microbial exposure to sulfamethoxazole and/or sulfamethazine has been shown to reduce microbial activity and/or inhibit extracellular enzymatic activities in soil (e.g., Liu et al., 2009; Frková et al., 2020). However, only a few authors have investigated the specific effects of these antibiotics on aquatic microbial community structure and functions (Yergeau et al., 2010, 2012; Johansson et al., 2014; Paumelle et al., 2021). Johansson et al. (2014) showed that sulfamethoxazole concentrations higher than 35 μg L^–1^ could trigger modifications in C source utilization in marine biofilm exposed for 4 days. Yergeau et al. (2010) showed that 8 weeks exposure to environmental concentrations of sulfonamides (0.5 μg L^–1^ of sulfamethoxazole or sulfamethazine) induced transcriptional changes in biofilm microbial communities. In a follow-up study, Yergeau et al. (2012) identified a negative impact of sulfamethoxazole on cyanobacteria abundance and on photosynthesis-related transcripts. Although sulfamethoxazole and sulfamethazine are structurally closely related and have the same modes of action, their effects on microbial communities are different in several ways (Yergeau et al., 2010, 2012).

Based on these findings, we made the following two hypotheses:

(1) Environmentally relevant concentrations of sulfamethoxazole and sulfamethazine will directly affect the bacterial structure (by exerting selection pressure toward resistant species) and functions (by reducing microbial activities) of periphytic biofilm.
(2) The ensuing changes to the bacterial community within the periphytic community will have indirect negative effects on the autotroph component.

To test these hypotheses, we investigated the effects of two levels of environmentally relevant concentrations of sulfamethazine (SMZ) and sulfamethoxazole (SMX), tested individually, on microbial activities and structure of natural periphytic biofilms cultivated in stream mesocosms for 28 days. The use of natural communities, such as periphytic biofilms, in controlled experiments is particularly useful for considering non-target species and biological interactions (Gessner and Tlili, 2016). This approach allowed us to increase the ecological realism of this ecotoxicological study. Microbial activities were assessed on a time scale by measuring functional parameters of the heterotroph (extracellular enzymatic activities, microbial respiration) and autotroph (photosynthesis) components of the biofilm. Structural effects were studied at the end of the experiment by assessing the taxonomic structure of diatoms and the genetics of bacterial communities. After 28 days of exposure, the main effects of both sulfonamides were observed on bacterial structure and on the diversity, viability and cell integrity of the diatom community. In particular, exposure to SMZ induced an increase in diatom teratologies at both concentrations. Thus, our study shows that sulfonamides can alter microbial community structure and diversity at concentrations currently present in the environment.

## Materials and Methods

### Biofilm Colonization

Natural biofilm was collected from the upstream Morcille river (Saint Joseph, 46°10′39.1″N 4°38′10.1″E) where the absence of any sulfonamide contamination in the sediment had previously been confirmed ([SMX] < 0.035 ng g^–1^; [SMZ] < 0.005 ng g^–1^). Biofilm was scraped from about 30 medium-sized stones and resuspended in river water before inoculation in aquaria to colonize glass slides (7.6 cm × 2.5 cm) for 22 days. Aquaria were filled with a mixture of demineralized water:groundwater (2:1 v/v, local groundwater used in previous experiments: Mahamoud Ahmed et al., 2018; Pesce et al., 2018) supplemented with K_2_HPO_4_ solution (0.1 mg L^–1^). A submersible pump (NEWA Jet 600) ensured water recirculation in each aquarium, which was exposed to a stable temperature (20°C) and natural light.

### Mesocosm Experiment

The experiment was performed in 15 glass indoor channels (length × width × height = 83 × 10.5 × 10 cm) previously described (Supplementary Figure S1; Mahamoud Ahmed et al., 2018). The bottom of each channel was covered with 28 glass slides previously colonized by biofilm and filled with 10 L of recirculating water (2:1 v/v demineralized water:groundwater). Flow rate and temperature conditions were similar to those described above. Light-emitting diodes (Radiometrix modules, total luminous flux 25116 lm) provided stable light conditions (12/12 h light/dark photoperiod). Light intensity was recorded every hour by a data logger (HOBO^®^ Pendant Temperature/light, Prosensor).

Three channels were exposed independently to one of the five different treatments tested:

1. Control: biofilm without antibiotic;
2. SMX: biofilm exposed to SMX at 500 ng L^–1^ = 126 μM;
3. SMX+: biofilm exposed to SMX at 5,000 ng L^–1^ = 1,266 μM;
4. SMZ: biofilm exposed to SMZ at 500 ng L^–1^ = 139 μM;
5. SMZ+: biofilm exposed to 5,000 ng L^–1^ = 1,391 μM.

These sulfonamide concentrations of 500 and 5,000 ng L^–1^ were chosen to reflect environmental concentrations (Deng et al., 2018). Antibiotic stock solutions of SMX (Sigma Aldrich, No-CAS: 723-46-6) or SMZ (Sigma Aldrich, No-CAS: 57-68-1) were prepared in water (2:1 v/v demineralized water:groundwater) and frozen at −20°C (final concentration: 16.5 mg L^–1^) before contamination.

To limit antibiotic loss by adsorption on the abiotic surfaces during the experiment, the experimental material was exposed to the corresponding concentrations of antibiotics for 4 days before the experiment started. Biofilm exposure to the five different treatments was then conducted for 4 weeks, and water was renewed every week using demineralized water:groundwater (2:1 v/v) phosphate-supplemented with K_2_HPO_4_ (final concentration 0.1 mg L^–1^) and contaminated or not by SMX or SMZ according to the treatment.

### Water Analyses

Physical and chemical parameters of water from each channel were measured each week before water renewal. Dissolved oxygen, conductivity, pH and temperature were quantified using portable meters (WTW, Germany). Water samples were collected to determine the concentrations of silicon dioxide (SiO_2_) according to NF T 90-007 and of chloride (Cl^–^), sulfate (SO_4_^2–^), nitrite (NO_2_^–^), nitrate (NO_3_^–^) and orthophosphate (PO_4_^3–^) ions according to EN ISO 10304-1.

Water samples (1 L) were also collected each week in each channel before water renewal and kept at −20°C before analyses of SMX and SMZ dissolved concentrations. The pH of a 100 mL water sample was adjusted to 4.0 ± 0.2 with HCl and 50 μL of 500 μg L^–1^ SMX-d_4_ and SMZ-d_4_ solutions (Toronto Research Chemicals and LGC standards, respectively) was added. The sample was then concentrated 200-fold on an Oasis HLB-200 mg cartridge (Waters^TM^) according to the manufacturer’s recommendations (elution solvent methanol). The SMX and SMZ concentrations were determined by LC/ESI-MS on a Thermo Scientific UHPLC Ultimate 3000 RSLC coupled to an Orbitrap Q-Exactive Analyzer. The analyses were carried out in positive mode. The UHPLC was equipped with a Luna Omega Polar C18 column; 100 × 2.1 mm; 1.6 μm (Phenomenex) at 30°C with acetonitrile gradient + 0.1% formic acid (Solvent A) and water + 0.1% formic acid (Solvent B): 0–2.5 min: 30–64.5% A (linear); 2.5–2.6 min: 64.5–99% A (linear); 2.6–5 min: 99% A; 5–5.1 min: 99–30% A; 5.1–8 min: 30% A, flow rate 0.45 mL min^–1^. For the mass spectrometer, gaseous N_2_ was used as nebulizer gas (50 A.U.). The spray voltage was 3.0 kV.

### Biofilm Sampling

Biofilm was sampled before exposure (Day 0) and after 7, 14, 21, 28 days of exposure. For this purpose, glass slides were collected before weekly water renewal and then scraped with a razor blade. The detached biofilm was suspended in a known volume of demineralized water:groundwater (2:1 v/v). On the day of sampling, the measurements of exo-enzymatic activities, respiration, photosynthetic yield and chlorophyll *a* concentrations were directly made on fresh biofilms. Biofilm suspension was kept either frozen to determine bacterial community structure or with preservatives to determine algal composition and diatom taxonomy as described below.

### Analyses of Microbial Community Functions

#### Respiration

Respiration was assessed under aerobic conditions on Days 0, 14, and 28 (Foulquier et al., 2013). When necessary, biofilm suspensions were diluted down to 2,000 μg of chlorophyll *a* L^–1^. 10 mL of biofilm suspension was then placed in a glass flask hermetically closed and incubated for 3 h, in the dark, under gentle agitation, at 20°C. Carbon dioxide production was measured by gas chromatography (490 MicroGC, Agilent Technologies) after 4 h and 24 h of incubation at ambient temperature (20°C). Respiration was expressed in nmol CO_2_ cm^–2^ h^–1^.

#### Exo-Enzymatic Activities

At Days 0, 7, 14, 21, and 28, potential activities of three extracellular enzymes (β-glucosidase EC 3.2.1.21, phosphatase EC 3.1.3.1, and leucine aminopeptidase EC 3.4.11.1) were measured by fluorimetry using substrate analogs coupled with a fluorochrome: MUF-Glu (4-methylumbelliferyl-β-D-glucopyranoside M3633 Sigma-Aldrich, CAS No. 18997-57-4) for β-glucosidase, MUF-Pho (4-methylumbelliferyl-phosphate M8883 Sigma-Aldrich, CAS No. 3368-04-5) for phosphatase, and MCA-Leu (L-leucine-7 amido-4-methylcoumarin hydrochloride L2145 Sigma-Aldrich, CAS No. 62480-44-8.) for leucine aminopeptidase (Romani et al., 2004; Pesce et al., 2018). Enzyme activities were measured for a range of substrate concentrations (0–3,000 μM for MUF-Glu and MUF-Pho, 0–2,000 μM for MCA-Leu) to ensure activity measurement at optimal substrate concentration. When necessary, biofilm suspension was diluted down to 650 μg L^–1^ of chlorophyll *a*. In a 96-well microplate, 70 μL of substrate and 150 μL of biofilm suspension were mixed and incubated at 15°C for 3 h 45 min. To stop the enzyme reaction, 20 μL of glycine buffer (0.05 M glycine, 0.2 M NH_4_OH, pH 10.4) was added. Fluorescence was then measured with a microplate reader (Synergy HT BioTek Instruments) with excitation wavelength 360 nm and emission wavelength 460 nm. To convert fluorescence values into enzymatic activities, standard curves of MUF (Sigma M1381 CAS No. 90-33-5) for β-glucosidase and phosphatase and MCA (Sigma A9891, CAS No. 26093-31-2) for leucine aminopeptidase were plotted at each time. Activities were expressed in nanomoles of hydrolyzed compound h^–1^ cm^–2^.

#### Chlorophyll *a* Concentration and Photosystem II Activity

At Days 0, 7, 14, 21, and 28, 3 mL of fresh biofilm suspension was used to estimate chlorophyll *a* (chl *a*) concentration and photosynthetic efficiency by multi-wavelength pulse-amplitude-modulated (PAM) fluorimetry using a Phyto-PAM instrument (H. Walz, GmbH) (Schmitt-Jansen and Altenburger, 2008). Characterization of photosynthetic efficiency is based on the measurement of maximum photosynthetic yield of photosystem II (Φ_*PSII*_). Biofilm samples were adapted for a few minutes to actinic light and two saturating pulses were done. Parameter Φ_*PSII*_ was calculated according to Genty et al. (1989)

$\Phi \,P\,S\,I\,I=\frac{F_{m}-F}{F_{m}}$ where *F*_*m*_ is the maximal fluorescence yield after the saturating pulse and *F* is the steady state of fluorescence.

### Analyses of Microbial Community Structure

#### Algal Community Composition

The enumeration of cyanobacteria, green algae and diatom cells was performed at Days 0, 14, and 28. On the day of each sampling, 4 mL of biofilm suspension was mixed with 150 μL of formol (37%) and stored at 4°C before analysis.

Samples were exposed to ultrasounds for 7 min for correct homogenization and 100 μL was placed in a Nageotte counting cell. Counting was done for 10 microscopy fields (200 cells minimum). Density was expressed in cells cm^–2^ (Morin et al., 2010).

Diatom species were identified as described in Morin et al. (2017) in the samples collected at Days 0 and 28 (*n* = 3 per treatment). Briefly, samples were exposed to boiling hydrogen peroxide (30%) and rinsed several times with distilled water. A few droplets were placed on slides and fixed with Naphtrax^®^ (AFNOR NF EN 13946). Over 200 frustules per slide were counted (i.e., >600 individuals identified per treatment) and diversity indices (specific richness, Shannon, evenness) were calculated.

#### Bacterial Community Structure

At Days 0, 7, 14, 21, and 28, 2 mL of biofilm suspension was centrifuged (6,500 × *g*, 2 min, ambient temperature) and after removing the supernatant, the pellets were stored at −80°C.

Total genomic DNA was extracted using FastDNA Spin kit for soil (MP Biomedicals, France) according to the manufacturer’s instructions. Evolution of bacterial community structure was assessed using a molecular fingerprint technique based on the polymorphism length of the intergenic transcribed spacer (ITS) between rRNA 16S and 23S gene. Amplification by PCR adapted from Ranjard et al. (2001) coupled with an automated ribosomal intergenic spacer analysis (ARISA) was carried out. Amplification of the ITS 16S-23S region was performed using primers 5′-6-carboxyfluorescein (FAM)-label ed-S-D-Bact-1552-b-S-20 (5′-TGCGGCTGGATCCCCTCCTT-3′) and L-D-Bact-132-a-A-18 (5′-CCGGGTTTCCCCATTCGG-3′) (Normand et al., 1996). The reaction medium contained, in a total volume of 50 μL: 5μL of 10× *Taq* reaction buffer (GE Healthcare), 200 μM of each deoxynucleotide, 0.5 μM of each primer, 5% dimethylsulfoxide, 0.5 U of rTaq DNA polymerase (illustraTM rTaq DNA Polymerase GE Healthcare), bovine serum albumin (Sigma, 0.3 mg.mL^–1^ final concentration) and 20 ng of DNA template. PCRs were obtained using a Thermal Cycler Tpersonal (Biometra, Göttingen, Germany) with the following program: initial denaturation at 94°C for 5 min, 35 cycles of denaturation (1 min at 94°C), annealing (1 min at 55°C), and extension (1 min at 72°C) and a final extension at 72°C for 10 min. DNA fragments were separated with sequencer ABI 3730xl (BIOfidal, Vaulx-en-Velin). An internal size standard G1200LIZ (20–1,200 pb) was used to normalize the data.

#### Quantification of Antibiotic Resistance Gene *sul1*

Before quantification of *sul1* and 16S rRNA sequence copy numbers, an inhibition test was carried out on each DNA sample as recommended by ISO 17601 (2016). qPCR reactions were carried out in a 15 μL reaction volume in a ViiA7 (Life Technologies, United States). For each reaction, the qPCR mixture contained 7.5 μL of Takyon MasterMix (Eurogentec, France), 1 μM of each primer [*sul1* (51F: AAATGCTGCGAGTYGGMKCA and 280R: AACMACCAKCCTRCAGTCCG, Wei et al. (2018); 16S rRNA primers according to Petrić et al. (2011)], 250 ng of T4 gene 32 (QBiogene, France), and 3 ng of DNA. The thermal cycling was performed as follows: 95°C for 10 min, 40 cycles of 95°C for 30 s, annealing at 60°C for 30 s and extension at 72°C for 30 s with data acquisition. A melting curve step was then run with 15 s at 95°C, 1 min at 68°C and then increase in temperature of 0.5°C s^–1^ to 95°C with data acquisition. Standard curves were obtained using serial dilutions of linearized plasmid containing sul1 amplicon. For each treatment, three independent replicates were analyzed. qPCR assays were duplicated. For each assay, three non-template samples were included. qPCR results were expressed as log of relative abundance of *sul1* sequence copy number per 16S rRNA copy number.

### Data Analyses

Statistical analyses were performed with R software version 3.4.4 (R Core Team, 2020). Normality of the residuals and data homoscedasticity were checked using Shapiro-Wilk (Royston, 1982) and Fligner-Killeen (Conover et al., 1981) tests, respectively. For each sampling time, differences in microbial parameters between treatments were estimated by analysis of variance (ANOVA) followed by a Dunnett *post-hoc* test. Results were considered significant if *p* < 0.05.

ARISA electropherograms obtained after sequencing were analyzed using Peak Scanner Software (Applied Biosystems). To eliminate background noise, an “optimal divisor” (Osborne et al., 2006) of 1/1, 000 was used. An abundance matrix of operational taxonomic units was obtained using the interactive binner script (Ramette, 2009).

For both diatoms and bacteria, abundance matrices were used to perform structure analyses. Distances between samples were represented by a non-multidimensional scaling (nMDS) based on a Bray Curtis dissimilarity index, using R package FactoMineR (Le et al., 2008). The stress value was calculated as an indicator of the agreement between the 2D projection and the predicted value from the regression following Clarke and Warwick (2001). To check whether the sample clustering observed on the nMDS plot was significant, an analysis of variance using distance matrices was performed (ADONIS test, 999 permutations, package Vegan: Oksanen et al., 2019).

## Results

### Physico-Chemical Parameters

Throughout the experiment, physical and chemical parameters in the channels remained stable and similar between treatments with mean values (*n* = 60) of 19.4 ± 0.3°C for water temperature, 7.9 ± 0.1 for pH, 180.5 ± 5.9 μS cm^–1^ for conductivity and 91.2 ± 1.3% for oxygen saturation. Most of these parameters were close to the characteristics of the sampling location in the Morcille river where pH was 7.3, oxygen saturation was 88.8%, and conductivity was 136.8 μS cm^–1^ on the day of sampling. However, the water temperature was 4 times colder in the natural environment in January (5.1°C). Nutrient levels were also similar between treatments with mean values (*n* = 20) of 2.30 ± 0.13 mg L^–1^ for silica, 2.78 ± 0.16 mg L^–1^ for nitrate and 0.06 ± 0.02 mg L^–1^ for phosphate in water before addition to the channels (data not shown).

### SMZ and SMX Concentrations

Sulfonamide concentrations in channel water varied according to the active substance and the level of contamination (Table 1). At Day 7, SMX concentrations were higher than SMZ ones for both the low (∼220 ng SMX L^–1^ vs. ∼157 ng SMZ L^–1^) and high (∼4,286 ng SMX L^–1^ vs. ∼2,780 ng SMZ L^–1^) contamination levels. However, whatever the sulfonamide and the contamination level considered, measured concentrations were lower than the expected ones (i.e., 500 ng L^–1^ and 5,000 ng L^–1^, respectively). In the high concentration treatments (SMZ+and SMX+), concentrations of both SMX and SMZ decreased continuously over time, with mean values of 2,783 ng L^–1^ and 960 ng L^–1^ at Day 28. SMZ and SMX concentrations were more stable in channels exposed to the nominal concentrations of 500 ng L^–1^ and were relatively similar for the two studied active substances at the end of the experiment (179.8 ± 13.6 ng SMX L^–1^ vs. 186.1 ± 104.0 ng SMZ L^–1^).

**TABLE 1:** Sulfonamide concentrations measured in the water of the channels.

Treatments	Sulfonamide	Expected concentrations (ng L^–^^1^)	Days	Measured concentrations (ng L^–^^1^)
SMX	Sulfamethoxazole	500	7	220.132.8
SMX	Sulfamethoxazole	500	14	204.121.6
SMX	Sulfamethoxazole	500	21	189.96.8
SMX	Sulfamethoxazole	500	28	179.813.6
SMX+	Sulfamethoxazole	5000	7	4285.8266.9
SMX+	Sulfamethoxazole	5000	14	4135.8310.7
SMX+	Sulfamethoxazole	5000	21	3189.474.7
SMX+	Sulfamethoxazole	5000	28	2783.0129.4
SMZ	Sulfamethazine	500	7	157.213.1
SMZ	Sulfamethazine	500	14	105.848.8
SMZ	Sulfamethazine	500	21	156.261.7
SMZ	Sulfamethazine	500	28	186.1104.0
SMZ+	Sulfamethazine	5000	7	2779.5582.9
SMZ+	Sulfamethazine	5000	14	1011.5192.7
SMZ+	Sulfamethazine	5000	21	1376.7313.9
SMZ+	Sulfamethazine	5000	28	959.7288.3

*Concentrations were measured every week of the 4-week experiment, before water renewal. Mean and standard deviations of measured SMX and SMZ concentrations (in ng L*^–^*^1^) are indicated (n = 3, per each treatment).*

### Heterotrophic Microbial Community

Sulfonamide contamination had few effects on the measured bacterial parameters, and most of the observed functional changes in response to sulfonamide exposure were transient.

Microbial respiration was similar between treatments and increased over time from 0.29 ± 0.09 nmol cm^–2^ h^–1^ at Day 0 (n = 3) to 14.88 ± 1.92 at Day 14 and 57.68 ± 11.10 nmol cm^–2^ h^–1^ at Day 28 (n = 15 channels, Supplementary Table S2).

Extra-cellular enzymatic activities exhibited various temporal trends independently of sulfonamide exposure (Table 2). The general trends observed were a decrease over time of microbial β-glucosidase activity (except at Day 14) and an increase over time of both phosphatase and leucine aminopeptidase activities. In unexposed biofilms (control), β-glucosidase activity was divided by almost 4 at the end of the experiment, while phosphatase activity was multiplied by 4 and leucine aminopeptidase by 6 (Table 2). In biofilms exposed to SMX or SMZ, β-glucosidase activity was mostly similar or lower than in unexposed biofilms. However, no significant difference (*p* > 0.05) was found between treatments (Table 2). During the first 14 days of the experiment, phosphatase activity was significantly lower (*p* < 0.05) in biofilms exposed to the sulfonamides tested than in unexposed biofilms (Table 2). In the last 14 days of exposure, phosphatase activity was slightly higher in biofilms exposed to the low and high concentrations of SMX than in unexposed biofilms (*p* < 0.05 at Day 21, Table 2). After 14 days of exposure, leucine aminopeptidase activity was almost 2 times higher in biofilms exposed to low SMX and SMZ concentrations than in unexposed communities (*p* < 0.05). However, this effect was transient, and no other significant difference was observed whatever the treatment or the sampling time (Table 2).

**TABLE 2:** Phosphatase and leucine aminopeptidase activities (nmol MUF cm^–2^ h^–1^) of microbial communities non-exposed (control) and exposed to 500 or 5,000 ng L^–1^ of sulfamethoxazole (SMX) or sulfamethazine (SMZ) over time.

Activity (nmolMUF.cm^–2^.h^–^^1^)	Days	Control	SMX	SMX+	SMZ	SMZ+
β-glucosidase	7	10.083.63	7.953.22	5.012.72	4.262.31	10.391.95
	14	7.560.55	9.431.04	5.220.95	8.242.71	7.351.92
	21	7.603.34	4.162.19	2.430.55	6.003.13	4.271.34
	28	2.661.54	2.441.76	2.231.91	1.150.05	1.220.14
Phosphatase	7	12.570.73	7.210.40*	11.020.84	6.181.45*	9.341.17*
	14	11.390.53	9.120.15*	12.131.03	9.111.01*	8.560.89*
	21	14.010.88	17.991.35*	17.420.93*	13.452.22	13.340.84
	28	57.3826.42	65.1020.19	66.7018.22	49.7812.13	43.798.13
Leucine aminopeptidase	7	3.470.83	3.290.39	3.440.20	3.960.42	3.370.42
	14	4.350.07	7.430.31*	4.170.37	9.081.97*	3.140.27
	21	13.682.91	13.282.67	15.961.11	16.185.61	9.812.64
	28	20.524.49	19.645.52	16.581.78	17.853.86	21.304.41

*Mean ± standard deviation of enzyme activities (nmol MUF cm*^–^*^2^ h*^–^*^1^) measured in the different treatments are indicated (n = 3 per treatment). Stars indicate significant differences between treatments and controls (Dunnett, p < 0.05).*

The antibiotic resistance gene *sul1* was detected in biofilms from all the treatments (including control). In all treatments, the relative abundance of sul1 was similar, with an average of 25.1 ± 5.3 *sul1* sequence copies per 1,000 16S rRNA sequence copy (*n* = 20, Supplementary Figure S2).

**FIGURE 1:**
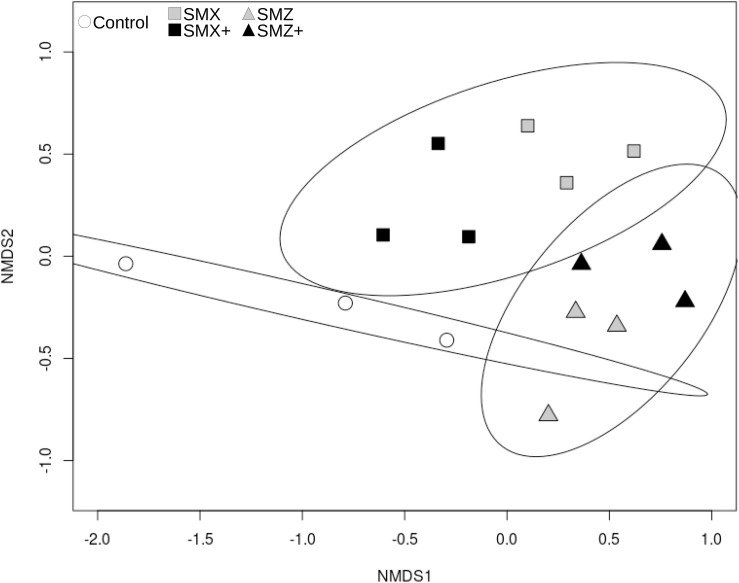
nMDS representation of Bray-Curtis distance similarities between bacterial communities based on ARISA after 28 days of exposure to sulfonamides (sulfamethoxazole, SMX; sulfamethazine, SMZ). The stress value is 0.143. Controls are represented by white circles, communities exposed to SMX by squares and to SMZ by triangles. The level of exposure to sulfonamides is color-coded as follows: gray for exposure to 500 ng L^–1^ and black for exposure to 5,000 ng L^–1^. Ellipses correspond to the 95% confidence interval around the centroid of the following groups: control, exposed to SMX (including SMX and SMX+), exposed to SMZ (including SMZ and SMZ+).

The ARISA performed after 28 days of exposure allowed retrieving 267 OTUs, bacterial community structure was slightly affected by the exposure to SMX and SMZ, at both the low and high concentrations (Figure 1). The permutational analysis of variance (ADONIS) shows that the exposure to sulfonamides explained 32% of the variance in the bacterial structure between samples (*p* < 0.01) and indicated a significant difference in bacterial structure between control biofilms, biofilms exposed to SMX and biofilms exposed to SMZ.

### Phototrophic Microbial Community

Sulfonamide exposure did not affect the effective quantum yield of photosystem II, which was relatively stable over time and between treatments (e.g., average yield 0.28 ± 0.05 at Day 28, Supplementary Table S1).

**FIGURE 2:**
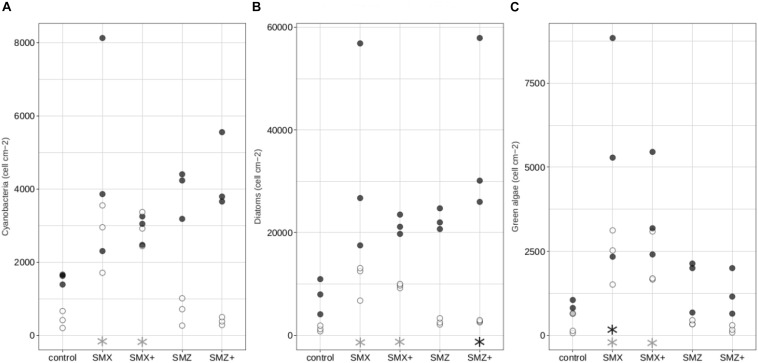
Composition of the autotrophic community: density in cells cm^–2^ of cyanobacteria **(A)**, diatoms **(B)**, green algae **(C)** in the control biofilms and biofilms exposed to sulfonamides. White circles correspond to biofilms exposed for 14 days while black circles correspond to biofilms exposed for 28 days. Significant differences (*p* < 0.05) between controls and biofilms exposed to antibiotics are indicated by gray stars after 14 days of exposure and by black stars after 28 days of exposure.

The relative proportions of the different algal groups (cyanobacteria, diatoms and green algae) were relatively constant over time and similar between treatments (Supplementary Figure S3). Nevertheless, cell densities of the different algal groups were different between treatments, with generally the highest cell densities observed in biofilms exposed to sulfonamides (Figure 2). Thus densities of cyanobacteria, diatoms and green algae were significantly higher (*p* < 0.05) in biofilms exposed to the low and high concentrations of SMX at Day 14 than in control biofilms (Figure 2).

All the microbial communities in biofilm were dominated by diatoms, whatever the treatment (Supplementary Figure S3 and Figure 2). Although sulfonamides did not have a clear effect on the structure of the diatom community (as illustrated in the nMDS plot in Supplementary Figure S4), exposure to sulfonamides led to a decrease in diatom specific richness, diversity and evenness, depending on the treatment (Table 3). Exposure to low and high concentrations of SMZ significantly reduced the specific richness and the Shannon index (*p* < 0.05) of the biofilm but no significant effect was observed on the evenness. Neither the low nor the high concentration of SMX had a significant effect on the specific richness, but exposure to the highest tested concentration of SMX significantly reduced both Shannon and evenness indices (Table 3).

**TABLE 3:** Structure and diversity of diatom community non-exposed (control) and exposed to 500 or 5,000 ng L^–1^ of sulfamethoxazole (SMX) or sulfamethazine (SMZ) after 28 days or exposure, represented by diversity index (specific richness, Shannon, evenness).

Diversity index	Control	SMX	SMX+	SMZ	SMZ+
Specific richness	20.671.08	16.671.47	19.002.55	15.000.00*	13.671.47*
Shannon	2.000.10	1.770.07	1.660.16*	1.640.07*	1.580.04*
Evenness	0.650.03	0.630.03	0.560.03*	0.610.03	0.600.01

*Mean ± standard deviation of diversity indices estimated in the different treatments are indicated (n = 3 per treatment). Stars indicate significant differences between treatments and controls (Dunnett, p < 0.05).*

**FIGURE 3:**
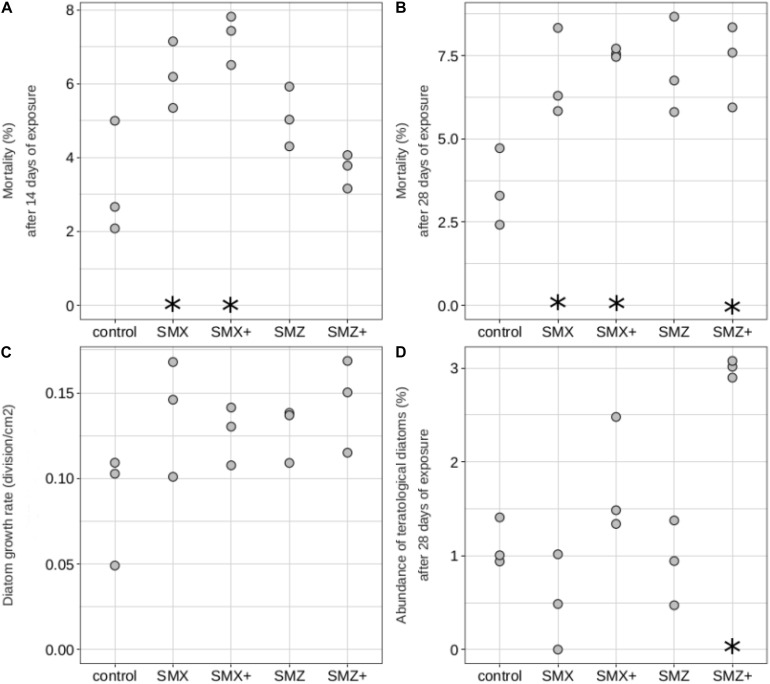
Diatoms mortality (in%, on Day 14: **A**, and on Day 28: **B**), diatom growth rate (in division cm^–2^, **C**), abundance of teratological diatoms (in%, on Day 28: **D**) of biofilms non-exposed (control) and exposed to sulfamethoxazole (500 ng L^–1^: SMX, 5,000 ng L^–1^: SMX+) or sulfamethazine (500 ng L^–1^: SMZ, 5,000 ng L^–1^: SMZ+). Significant differences (*p* < 0.05) between controls and biofilms exposed to antibiotics are indicated by stars.

The physiology and morphology of diatoms were also affected by sulfonamides (Figure 3). At Days 14 and 28, diatom mortality was significantly higher in biofilms exposed to SMX than in control biofilms (*p* < 0.05, Figure 3A) and a similar trend was observed at Day 28 for biofilms exposed to both concentrations of SMZ (significant result only for biofilm exposed to 5,000 ng L^–1^, Figure 3B). Diatom growth rates were also slightly higher in biofilms exposed to sulfonamides than in control biofilms despite the lack of significant difference (Figure 3C). In biofilms exposed to the highest sulfonamide concentrations tested, we observed an increase in the abundance of diatoms exhibiting deformities, in particular the abundance of teratological diatoms was more than 2 times higher in biofilms exposed to SMZ (reaching 3%) than in control biofilms (1.1%).

## Discussion

Water contamination by environmentally realistic concentrations of SMZ and SMX affected both the heterotrophic and autotrophic communities of biofilms with various effects according to the sulfonamide and to the exposure level. Supporting our hypotheses, exposure to SMX, and to a lesser extent to SMZ, modified bacterial structure and impaired microbial enzyme functions. Moreover, sulfonamide exposure also had negative effects on the autotroph component of periphytic biofilm.

Concerning the heterotrophic component of the biofilm, both sulfonamides modified phosphatase and leucine aminopeptidase extracellular enzyme activities in the first weeks of exposure, with no effect on β-glucosidase activity. Nevertheless, these effects were transient: after 4 weeks of exposure, all the measured extracellular enzymatic activities recovered and were similar between control communities and communities exposed to sulfonamides. Together with this functional recovery, small changes in the structure of the bacterial communities exposed to sulfonamides were also observed. These results suggest first a direct effect of the sulfonamides on the exposed communities, resulting in impairment of the bacterial functions as already observed in soil microbial communities exposed to SMX (Liu et al., 2016) or in biofilm exposed to river water strongly contaminated by pharmaceutical residues including antibiotics (Proia et al., 2013a). In addition, the functional recovery observed after 4 weeks of exposure, together with the changes in bacterial community structure, suggests that sulfonamide exposure acted as a selection pressure on the microbial communities, selecting the most tolerant species able to maintain the reference level of extracellular enzyme activities on exposure to sulfonamides. Previous studies highlighted the impact of environmentally realistic levels of antibiotics, including sulfonamides, on the bacterial structure in soil (Liu et al., 2016), sediments (own unpublished results), or biofilm (Proia et al., 2013a).

In our study, bacterial structural changes following sulfonamide exposure were not associated with a higher relative abundance of the *sul1* resistance gene in microbial communities exposed to sulfonamides than in controls. The level of this antibiotic resistance gene remained stable throughout the experiment. This result is in agreement with the observation of Subirats et al. (2018) who found no significant difference in the relative abundance of *sul1* genes between control biofilms and biofilms exposed to a mixture of five contaminants, including sulfamethoxazole, at 0.1 mg L^–1^ each (methylparaben, ciprofloxacin, diclofenac, erythromycin and sulfamethoxazole) for 28 days in mesocosms. To go beyond the community capacity to resist antibiotics (assessed here by the relative abundance of *sul1* gene in the total bacterial community) and to validate the above hypothesis of the selection of the most tolerant species, it would be of interest to assess the functional resistance of the community to the acute toxicity of antibiotics through pollution-induced community tolerance (PICT) measurements based on short-term toxicity tests (e.g., Corcoll et al., 2014).

Concerning the autotrophic component of the biofilm, the exposure to both sulfonamides led to an increase in cell density and diatom mortality with no impact on the autotrophic community structure based on the relative distribution of the main groups (i.e., diatoms, cyanobacteria and green algae). This stimulatory effect of low concentrations of SMX on algal growth has been previously observed in marine biofilms exposed to SMX (Johansson et al., 2014) and may be a consequence of temporarily decreased fitness of the heterotrophic compartment (e.g., Proia et al., 2011). The higher cell density observed in biofilm communities exposed to sulfonamides in our experiment is therefore likely to be due to the presence of tolerant species with high competitive abilities. The higher mortality values in these communities may reflect the presence and elimination of the sensitive species following SMX/SMZ exposure.

Sulfonamides did not affect only microalgal growth dynamics but also diatom morphology, since the percentage of teratologies was higher in communities exposed to the highest concentration of sulfonamides tested than in non-exposed ones. Metals are known to induce deformities in diatoms (e.g., Lavoie et al., 2017; Tiam et al., 2019), but to our knowledge, this is the first report of a teratogenic effect of antibiotics on diatoms. There is no evidence in the literature that sulfonamides can directly cause diatom deformities, and the teratogenic effects of sulfamethoxazole exposure observed in our experiment could be due to indirect effects. The higher diatom mortality and cell density in exposed communities than in non-exposed ones suggest a higher renewal rate of diatom cells in those exposed communities. This higher renewal rate could be the result of greater growth rates of teratological individuals over normal ones (Coquillé and Morin, 2019).

The observed effects of sulfonamides on non-target species, such as diatoms, may also result from interactions between microalgae and bacteria within the periphytic biofilm. Indeed, previous studies have highlighted the tight link between these two complementary groups (e.g., in the carbon cycle: Battin et al., 2016), and the repercussion of direct toxic effects on one group to another (non-target) one (e.g., after exposure to the bactericide triclosan: Proia et al., 2011). The direct effect of sulfonamides on bacteria may also change the heterotrophic community composition and the quality of bacterial exudates for microalgae and then lead to alterations in the morphology and survival of diatoms. Since Windler et al. (2014) showed that the absence of essential accompanying bacteria in laboratory cultures induced teratologies in diatoms, we can hypothesize that the structural changes in bacterial structure induced by sulfonamides led to the observed increase in diatom deformities. Further experiments are needed to better understand how sulfonamides might modulate microalgae-bacteria interactions within biofilms.

Altogether, our results highlight that even low concentrations of antibiotics can modify, at least transiently, microbial community structure and functions.

## Conclusion

Sulfamethazine and sulfamethoxazole had similar effects on biofilm communities after 4 weeks of chronic exposure to nominal concentrations of 500 and 5,000 ng L^–1^, with exposure to the higher concentration generally resulting in stronger effects. Exposure to sulfonamides mainly affected bacterial structure within the heterotrophic component of biofilm. In the autotrophic compartment, those two antibiotics particularly affected the dominant algal component (diatoms), by increasing their mortality and inducing teratologies at the higher of the two concentrations tested. Our study shows that sulfonamides are susceptible to alter microbial community structure and diversity at concentrations frequently found in the environment. In particular, non-target species such as diatoms are likely to be affected by contamination by sulfonamides, with unknown functional consequences for the ecosystem.

## Data Availability Statement

The raw data supporting the conclusions of this article will be made available by the authors, without undue reservation.

## Author Contributions

CB and SP designed the experiment with the help of LK and AR. LK and AR led the experimental work during mesocosm exposure. PB-H, LK, and ML did the chemical analyses of sulfonamides. MM did the analyses of major ions in water surface. LK, AR, and CB did the functional microbial analyses. LK did the DNA extraction. FM-L, MD, and JB did the quantification of *sul1* genes. SM did all the analyses of algal community composition. LK and CB did the data treatment, statistical analyses, and wrote the first version of the manuscript. SP, FM-L, PB-H, and SM reviewed, completed, and improved the first version of the manuscript. All authors contributed to the article and approved the submitted version.

## Conflict of Interest

The authors declare that the research was conducted in the absence of any commercial or financial relationships that could be construed as a potential conflict of interest.
