# Supplementary material for: Environmental Concentrations of Sulfonamides Can Alter Bacterial Structure and Induce Diatom Deformities in Freshwater Biofilm Communities
Source: Front Microbiol. 2021 May 7;12:643719. doi: 10.3389/fmicb.2021.643719 (PMC8137839; doi:10.3389/fmicb.2021.643719)
Supplement: Supplementary file 1 [file Presentation_1.pdf]

Environmental concentrations of sulfonamides can alter bacterial structure and induce diatom deformities in freshwater biofilm communities

Laura Kergoat, Pascale Besse-Hoggan, Martin Leremboure, Jérémie Beguet, Marion Devers, Fabrice Martin-Laurent, Matthieu Masson, Soizic Morin, Amélie Roinat, Stéphane Pesce, Chloé Bonnineau

SUPPLEMENTARY MATERIAL

Table S1. Effective quantum yield in microbial communities non-exposed (control) and exposed to 500 or 5000 ng L^{-1} of sulfamethoxazole (SMX) or sulfamethazine (SMZ) over time. Mean ± standard deviation of effective quantum yield (r.u.) are indicated (n = 3 per treatment).

	Days	Effective quantum yield
Control	7	0.17 ± 0.04
	28	0.32 ± 0.07
SMX	7	0.23 ± 0.04
	28	0.29 ± 0.06
SMX+	7	0.23 ± 0.06
	28	0.26 ± 0.02
SMZ	7	0.24 ± 0.06
	28	0.25 ± 0.03
SMZ+	7	0.21 ± 0.03
	28	0.26 ± 0.03

Table S2. Microbial respiration (nmol CO2 cm^{-2} h^{-1}) measured after 4h of incubation in microbial communities non-exposed (control) and exposed to 500 or 5000 ng L^{-1} of sulfamethoxazole (SMX, SMX+) or sulfamethazine (SMZ, SMZ+) over time. NA: initial microbial communities sampled before contamination.

Sampling day	Treatment	Microbial respiration (nmol CO2 cm^{-2} h^{-1})
0	NA	0.33
0	NA	0.36
0	NA	0.18
14	Control	14.74
14	Control	16.03
14	Control	14.35
14	SMX	16.97
14	SMX	15.19
14	SMX	16.06
14	SMX+	17.51
14	SMX+	16.92
14	SMX+	12.08
14	SMZ	12.28
14	SMZ	15.45
14	SMZ	14.71
14	SMZ+	11.63
14	SMZ+	16.60
14	SMZ+	12.77
28	Control	47.82
28	Control	62.73
28	Control	57.28
28	SMX	53.81
28	SMX	61.41
28	SMX	79.09
28	SMX+	63.50
28	SMX+	78.81
28	SMX+	60.94
28	SMZ	43.27
28	SMZ	52.04
28	SMZ	60.95
28	SMZ+	47.35
28	SMZ+	54.39
28	SMZ+	41.77

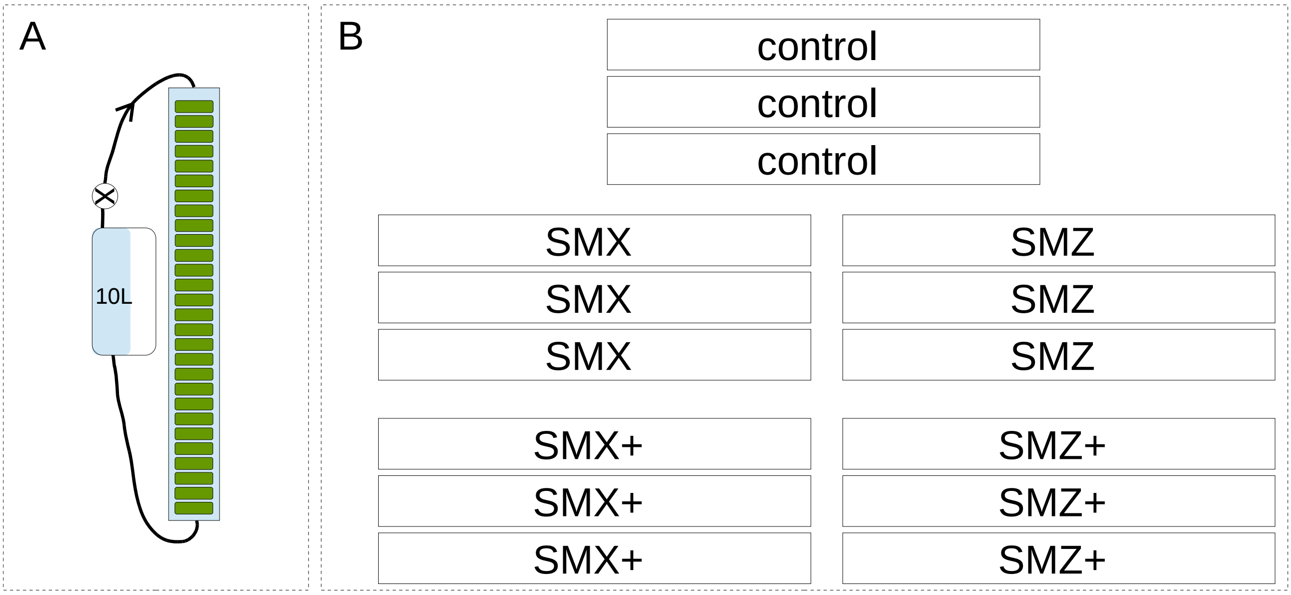

Figure S1. A: schematic drawing of an experimental channel. B: experimental design, SMX: channels exposed to 500 ng L^{-1} of SMX, SMX+: channels exposed to 5000 ng L^{-1} of SMX, SMZ: channels exposed to 500 ng L^{-1} of SMZ, SMZ+: channels exposed to 5000 ng L^{-1} of SMZ.

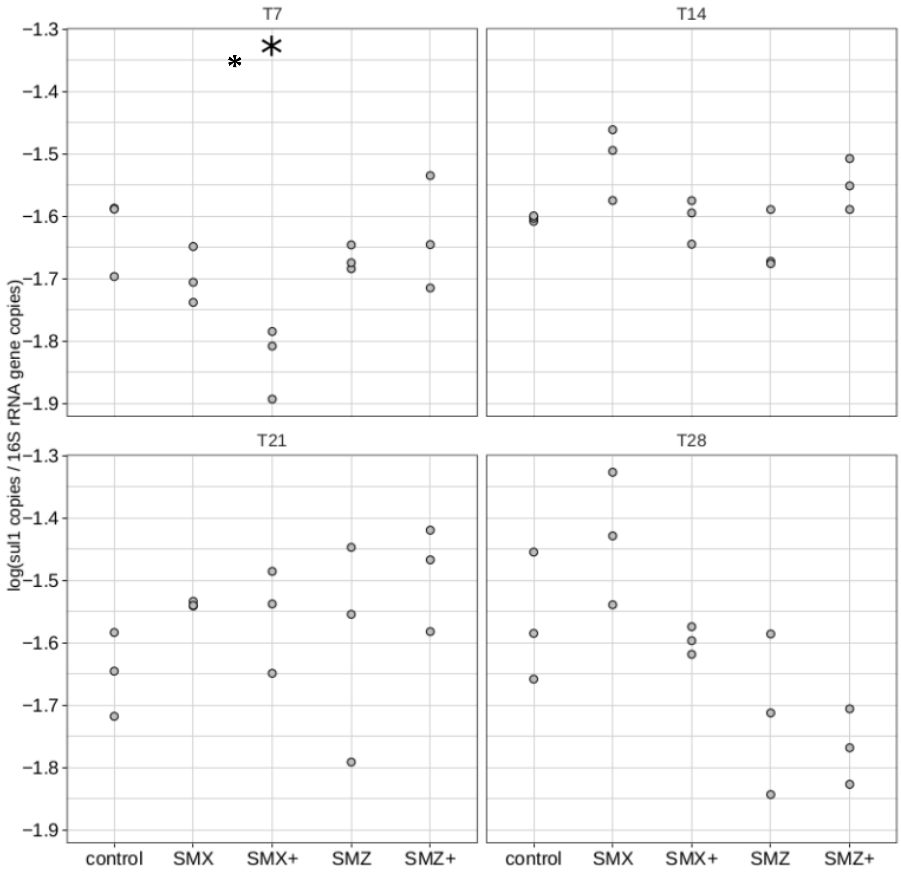

Figure S2. Relative concentration of *sul1* in control biofilms and in biofilms exposed to sulfamethoxazole (500 ng L^{-1}: SMX; 5000 ng L^{-1}: SMX+) or sulfamethazine (500 ng L^{-1}: SMZ; 5000 ng L^{-1}: SMZ+) over time. Each point corresponds to one of three different biological replicates. Exposure time is indicated above each graph: 7, 14, 21 or 28 days. Stars indicate significant differences ($p < 0.05$, Dunnet test) from controls of biofilms exposed to sulfonamides.

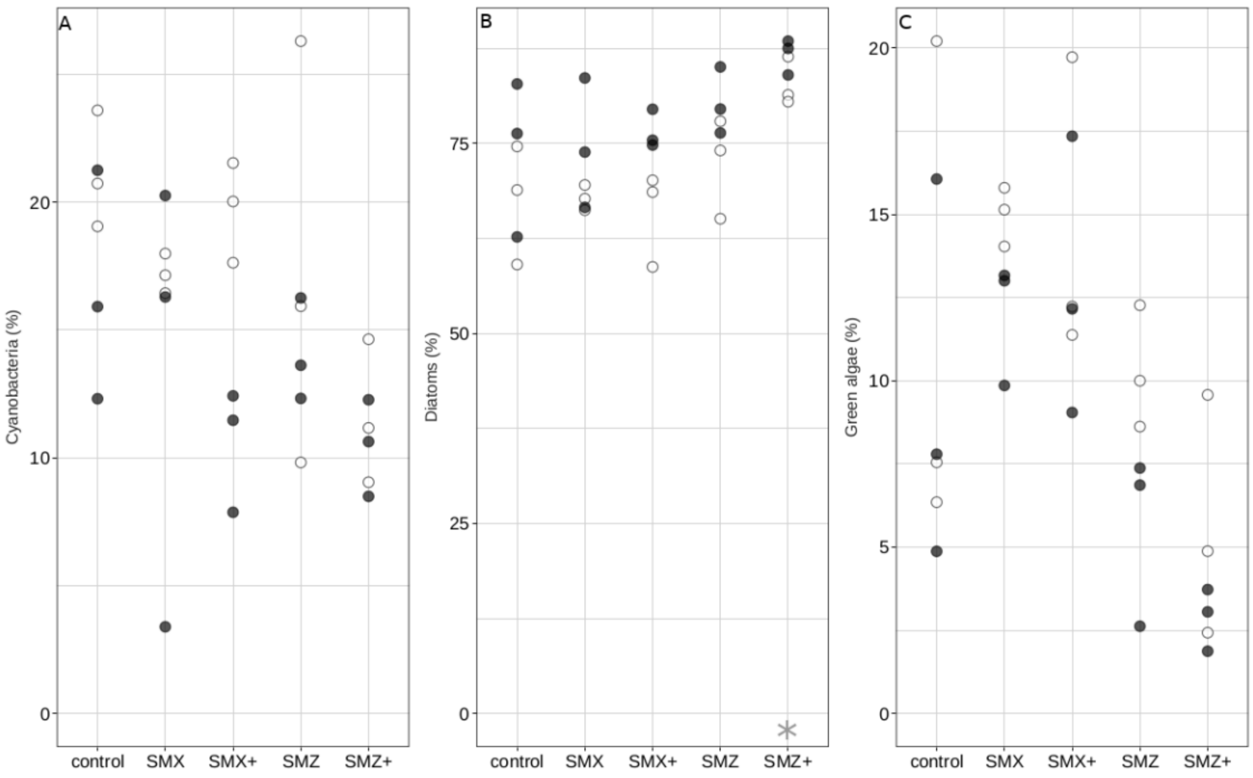

Figure S3. Composition of the autotrophic community: percentage of cyanobacteria (A), diatoms (B), green algae (C) in control biofilms and biofilms exposed to sulfonamides (500 ng/L: SMX, 5000 ng/L: SMX+; 500 ng/L: SMZ, 5000 ng/L: SMZ+). White circles correspond to biofilms exposed for 14 days while black circles correspond to biofilms exposed for 28 days. Stars indicate significant differences ($p < 0.05$) from controls of biofilms exposed to antibiotics after 14 days of exposure.

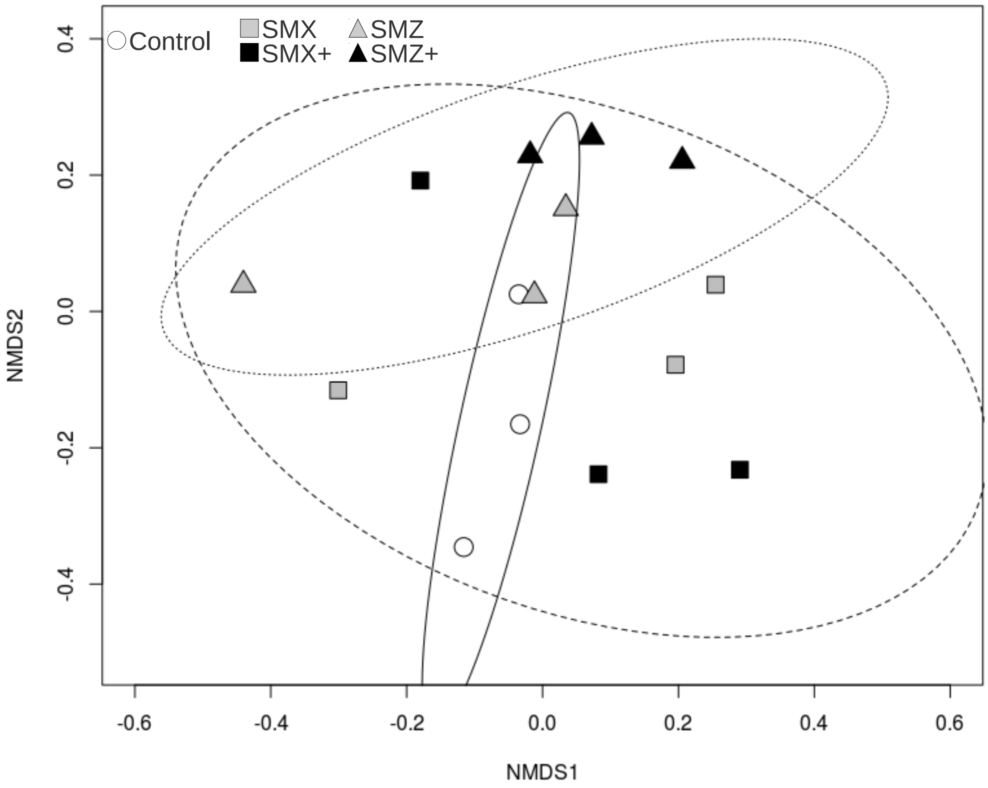

Figure S4. nMDS representation of Bray-Curtis distance similarities between diatom communities after 28 days of exposure to sulfonamides (sulfamethoxazole, SMX; sulfamethazine, SMZ). The stress value is 0.192. Controls are represented by white circles, communities exposed to SMX by squares and to SMZ by triangles. The level of exposure to sulfonamides is color-coded as follows: grey for exposure to 500 ng L^{-1} and black for exposure to 5000 ng L^{-1}. Ellipses correspond to the 95% confidence interval around the centroid of the following groups: control, exposed to SMX (including SMX and SMX+), exposed to SMZ (including SMZ and SMZ+).
